# Challenges for the understanding of the dynamics of social coordination

**DOI:** 10.3389/fnbot.2013.00018

**Published:** 2013-10-11

**Authors:** Julien Lagarde

**Affiliations:** Movement to Health Laboratory, EuroMov, Montpellier 1 UniversityMontpellier, France

**Keywords:** coordination dynamics, perception–action coupling, asymmetric roles, creation of information, taxonomy

## Abstract

The way people interact can be examined by looking at the way they move relative to each other. Seeking the principles behind those interactions have consequences potentially related to any type of interpersonal function, far beyond the so-called “motor” processes typically associated with the study of movements, be it perceptive, cognitive, affective, pragmatic, or epistemic. Here, we present the way the framework of coordination dynamics define and addresses the interactive actions in a dyad. We first introduce the basics of pattern formation as the roots of the theoretical approach of coordination dynamics, and then the way this framework may contribute to establish a solution to classify behaviors. Thereafter we review promising empirical results on the dynamics of interpersonal coordination, and finally discuss were to go next to decipher the way the coordination between two people and the way each individual contribute may be disentangled.

## INTRODUCTION

The way the current results of research on social coordination belonging to the framework of coordination dynamics are presented and discussed in this paper is the outcome of numerous interactions with close collaborators over the past years. At the same time the author cannot escape being the sole responsible for every statement written here. The present paper aims at identifying essential solutions and outstanding challenges in understanding the interaction between people from within the theoretical and experimental framework of the coordination dynamics approach. However, to begin with, in a provocative and hopefully not too unusual tone, I will dwell in here a little by developing this basic standpoint of the author. These two first sentences above introduce the idea of an individual~others couple, which, even if it may appear far-fetched, relates to the general purpose of this paper and in particular to the discussions reached in its final part. Taking a short cut^1^ they address the question about the separation between the author and its collaborators.

This separation may come to asking where “I” do start and end, how do I know who I am without an observer and by definition perturbing eye, could it be said that I exist outside my relation to the other(s), or here is another one: what is different between you and me, I mean really different? Descartes aimed at solving a related question exerting his doubt to decide what could ground the very possibility of his existence. Within the boundaries he chose to define the problem and the method, nothing could resist his own doubt but that he was actually thinking. The fact that he was thinking provided him with a proof about his own existence, no less and no more. It is beyond the scope of the present paper of course to analyze the philosophical validity of this demonstration, and also to present the many challenging and disputed views this rationale triggered. It is said by many however that the famous cogito gave shape and momentum to deleterious conceptions, giving rise to some sort of separation between the brain, the body, the mind. As neurosciences evolve it becomes more and more clear that understanding the relation between those entities is still among the most ambitious enterprises. One could add on the list the understanding of the relations between the individual and its environment, physical and social. Anyway on the long run, I wonder whether considering “I am” as Monsieur Descartes did will prove the right starting point to define the existence of homo sapiens, or coming back to a scientific level of analysis, to understand the lawfulness of his/her behavior, and of the functioning of his/her brain. Therefore one may include individual and others in the realm of things difficult but necessary to relate; that might even be a prerequisite to understand some basic cognitive functions, beyond the one related to the large class of communicative acts. Here I will present a framework that is involved in the search for basic understanding of the relation between humans, starting from the relation between their movements. However this framework is not restricted to movement generation and control understood as “motor,” it has implications for perception, cognition, rehabilitation of the so-called social disabilities, and learning.

## THE FRAMEWORK OF ELEMENTARY COORDINATION BEHAVIOR

Incredibly complex systems like a performing athlete display a high degree of spatial and temporal order between its components; hence it may be essentially captured by composite, higher order variables. It has been shown that some of these variables are not mere post hoc idealizations; they follow the tendencies of the various components (limbs, muscles) to organize their motions in relation to each other and form patterns of behavior (Turvey, 1990; Kelso, 1995). Coordination is often said to be the rule and not the exception in biological systems, and is surely leading the game in perceptivo-motor problems we solve every half a seconds in our daily life. The most obvious such patterns, because they belong to overt directly measurable behaviors, originate in interlimb coordination (walking, standing, reaching, chewing, and speaking). Those patterns are best described by temporal, spatial, or forces and torques relations, they share the very helpful characteristic of being much lower dimensional than the multitude of the components they gather.

Many authors have contributed to develop a theoretical and empirical framework to explain how coordination patterns arise, since the pioneers (Bernstein, 1967; Kugler et al., 1980), to the most modern developments (Kelso, 1995; Haken, 1996; Jirsa and Kelso, 2004), notably assuming a key role for self-organized emergence in brain and behavior. Most of the time, this framework is referred to as coordination dynamics. It addresses coordination between joints, between limbs and environment, like the synchronization to a beat, and more recently the coordination between people.

Basic ingredients of the framework are the following: components are interacting via couplings, the couplings cause the increase of the order between the components, up to the stabilization of patterns. The components can be joints, also muscles, and the patterns are very often, but not restricted to in principle, timing relations between joints movements. Sources of couplings are manifold. It can be functional exchanges between neural assemblies, interaction torques between joints, or of a perceptual basis, consider for instance how vision can provide relative information when I try to put a thread through a needle’s hole. The most well understood elementary coordination is bimanual coordination. When asked to oscillate the index fingers people are able to establish and maintain two patterns of motion, either flexing and extending simultaneously the two fingers, or in opposite way. The first pattern, sometimes described as mirror movements, is measured by a phase difference close to 0 radians, in-phase, while the second is measured by an anti-phase difference (pi radians). When the rate of movement is increased only the in-phase pattern can be maintained, if intended, the anti-phase pattern is spontaneously abandoned and the in-phase is adopted instead. The way this change operates has deep theoretical consequences. Haken et al. (1985) assumed that those coordinations obeyed the laws of pattern formation, designed originally for large scale systems in statistical physics. They predicted that the change of pattern corresponded to a phase transition encountered in physics, and thus should operate by a loss of stability of the intended anti-phase pattern. This prediction has been verified experimentally, and further developments taking into account biological noise led to stochastic predictions (e.g., critical fluctuations, first passage time, correlations), and again to converging evidence (Schöner et al., 1986). This initial round of theoretical predictions and crucial experiments, exotic as it was at the time in this field, shake the theory of biological control of movement inspired by cybernetic and computer program metaphors. Self-organization can work. Additional astonishing support for the validity of this approach comes from related experiments in bimanual coordination, this time examining the organization behind the coordination of index fingers moving at different frequencies (Kelso and DeGuzman, 1988; Beek, 1989; DeGuzman and Kelso, 1991; Peper et al., 1995). The stable frequency ratios between left and right hand oscillatory movements that a human can establish correspond quite closely to the famous Arnold’s tongues discovered for celestial mechanics. Those ratios belongs to the set of rational number corresponding to quotient of integers; a seemingly wild biological zoo however well predicted by the one-dimensional circle map model.

**FIGURE 1:**
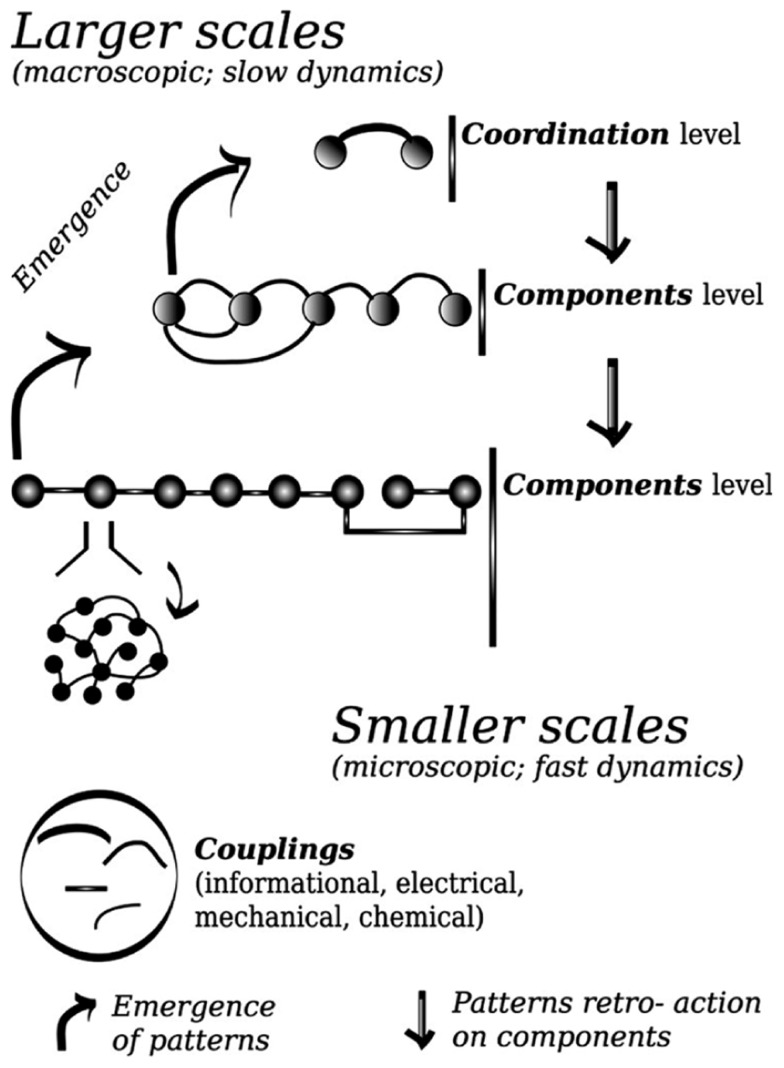
**Schematic representation of the principle of decomposition between coordination variables and components variables.** Coordination patterns emerge from the cooperation between components of a smaller scale; once formed these patterns constraint the evolution of lower scales (see Kelso, 1995; Haken, 1996). Couplings among components determine the cooperation. In the lower panel the meaning of the graphical signs used in the higher panel is presented.

In the same vein as in statistical physics, the patterns arise from interacting components. Those comprise minimally here the individual’s finger movements, but also the muscles, and spinal–brain neural ensembles related to each finger. One may think about components in terms of functional units, which can operate at various scales. The patterns are low dimensional, in that they require one or few coordinates to be described, that is, to define the state space onto which their dynamics can unfold. The dynamics can then be tracked down and modeled at the level of the patterns. Like in previous modeling of phase transitions, there is a deep relation between the high dimensional behavior of the system taken as a whole, and the low dimensional evolution of the patterns. The components are said to be “enslaved” by the patterns; approaching of the tipping point of change the pattern is losing its stability, its dynamics slows down, while the components are kept stable. A stable state possesses fast dynamics, practically a short relaxation time. These changes and contrast of stability impose a separation of time scales, which confer to the slowly evolving pattern the lead of the whole dynamics (see **Figure 1**). Those properties are generic around bifurcations in low dimensional dynamical systems, and correspond to the operation of the center manifold theorem, widely used to reduce the dimension of large dimensional problems to make then tractable.

The mapping of the dynamics onto those low dimensional attractors remains non-intuitive for many when applied to intentional systems like animals or humans. The friendly skeptics reduce this phenomenological modeling to a default practical solution to an otherwise intractable problem. This is a clear misunderstanding. We are beyond a practical way out complexity: the patterns are real, in that their stability is real and can be directly measured experimentally, and their formation is thus real, as much as anything else in science can be. This does not mean that the laws of coordination are not abstract, as we would see later, but to me real and abstract are two completely incommensurate properties. The proponents of so-called materially grounded models often end up relying on a mechanical level of description. Why not if the empirical evidence calls for it and the corresponding driving theory pushes us forward, but it is to my understanding completely misguided to conceive mechanical laws as less abstract than any other. Are not the conservation laws explained by abstract symmetry properties, demonstrated by the famous Noether’s theorem?

The emphasis in this framework is given to the formation of those patterns. It entails that a pattern of behavior has to be, by the same mechanisms, established and maintained continuously, in particular to resist external perturbations or internal biological noise. There is another contribution to disorder, apart from noise, with which we will end up this introductory short course on coordination dynamics. The establishment of pattern has to oppose a very general contribution to disorder: the asymmetry of components. Some components can be faster than others for instance, or have more inertia than others. This asymmetry, again this reasoning is grounded on very generic principles, leads to a reduction of stability and eventually requires to be opposed by stronger coupling to maintain the pattern. They may well lead to a brake down of the pattern, then to a change to another pattern if available and goal relevant, or to disorder, error, and failure of any kind. But think about it, in a system like a brain, if homogeneity was the rule, then trivial ensemble synchrony would be mandatory, and not much could arise apart from epileptic seizure. If the same logic was applied to groups of individuals, I guess we would also form a boring crowd, may be identically lethal, for we would be too similar to each other.

As a supplementary note, it is wrong to confine those processes to low level brain mechanisms, like “motor system,” or to bottom up, or unconscious, or automatic processes. This approach can be widely applied to a variety of functions, and is much more specific then a “bottom up” type of brain processes.

## A CLASSIFICATION OF BEHAVIORS

A key unresolved issue in behavioral sciences and neurosciences is to classify tasks and more importantly related behaviors. We use various tasks in our experiments, obtain similar or seemingly distinct results, but we lack a fundamental classification tool, in the same vein as the classification of atomic elements by Mendeleev. Without this breakthrough, we cannot even clearly generalize ours results to some class or set of behaviors, or understand why two experiments studying apparently similar processes failed to get identical results. Some propose that processes are task specific; however such a position is aimless until unequivocal principles to sort those tasks would be available. Once further advanced, this issue will not represent anymore such a crucial limitation to our understanding of individual and interpersonal behaviors. Basically a researcher may find a slight value in understanding what he/she means by using the words “distinct,” or “similar.” This may sound overly provocative, but past and current research faces a real issue right here. In this section some steps toward a clarification are presented, though dramatically incomplete respective to the grand challenge faced.

What are the variables controlled by the central nervous system (CNS)? Note that what is meant here by the utterance “variables controlled,” despite its very common use, depends on what is your preferred theoretical inclination, it could be understood either directly in a control theoretic, cybernetic framework, or with a different flavor, according to a theory of emergence of patterns. In the latter one may speak about “control without a controller,” and of “effective variables,” typically the ones defining the patterns, hence the variables for which the current intended function requires stabilization. Those are the variables that bifurcate when a control parameter is varied, from disorder to order or vice versa, or between states in multistable dynamics (walking–running).

Gentile (1998) distinguishes various stages during learning, here what may be to retain is “the content of each stage,” meaning what is learned. She proposes that firstly the “topology” of the movement is acquired, defined as the gross spatial pattern, using the intuitively formulated concept of invariance, pervasive to the study of biological movement, since at least Bernstein (1967). At a second stage she identifies the fine tuning of force, mainly understood at the joint level (torques). The acquisition of a spatial pattern precedes the fine tuning of forces, hence is assumed a time hierarchy between goal successes enabled by the acquisition of the gross approximation of coordination and by fine control. This distinction overlaps with another one, which distinguishes the acquisition of a spatial pattern of the movement of the end-effector from the acquisition of fine control at the joints level. Note that most often the end-effector is concerned directly with the task’s goal, whether it is in terms of spatial or temporal accuracy, it may also includes (Newtonian) dynamics requirements (forces, compliance) especially when the physical interaction with objects is involved. Please note that a similar distinction has being framed in ideomotor theories, which states that the effect of actions, or final goal, but not the actual effectors is relevant for the control (Hommel, 1993; Prinz, 1997). Another classification that seems helpful was proposed by Schöner (1995). He distinguishes tree levels of variables which may be controlled differently by the CNS. Based mainly on perturbation studies, aiming at finding the invariant properties of movement, he distinguishes between the timing level, the force (“loads”) level, and the goal level. Timing refers to the relative time structure between the limbs motions, hence a relative phasing, which is applicable to both continuous-rhythmic movements and discrete ones. The timing class also includes timed movements in relation to the environment, for instance in catching a base ball flying ball, avoiding an obstacle, rowing in synch with teammates. The load level refers to the invariants properties with respect to force production, found with loading perturbation studies, notably following Feldman (1980) formulation of the equilibrium point theory. The goal level points at the variables which capture the effect the movement should produce be it a spatial, temporal, or force outcome. In the self-organization pattern formation framework, the first move was to distinguish and relate the level of the components and the level of the coordination, which we already presented briefly above as a basis for our methodology of selection. The basic empirical evidence and theoretically grounded argument is that robust effects at the coordination level, concerning essentially dynamics, can be obtained irrespective to a large set of changes of the components. This means specifically the same type and number of stables patterns, here, phase relations, can be formed and maintained, and the changes by bifurcation between them, when a parameter is varied (frequency), are kept invariant despite changes in the components. Hence the components may differ, but the only way to get the same coordination dynamics is that the couplings between these components share some invariance.

In the above attempt of classifications, invariance is the key. One aims at finding what is left invariant after applying a transformation (perturbation, change of components, coordinate change, projection, mapping), or a group of such transformations. As we will see a bit further in the next classification, dynamical systems, here applied to human skilled behavior, naturally make use of tools to define and detect invariance, for instance when identifying states and bifurcations.

## THE CASE FOR TOPOLOGICAL EQUIVALENCE

Jirsa and Kelso (2005) used the definition of topological equivalence, defined in dynamical systems, to rigorously define what makes a difference qualitative and not only quantitative. They studied simple movements, periodic or discrete at the end-effector level of individual limb. To get a tractable problem, they studied a case with only two dimensional dynamics; hence it corresponds to what is called a two dimensional phase flow. When two classes define two qualitatively distinct skills, the intersection between the sets of these two classes is necessary empty. Moreover, there exists a type of mapping, defined under topological rules (to keep it very simple: one can stretch, bend, twist but not cut; topology in this domain is sometimes pictured as the “rubber sheet” geometry), within elements of one class but not between elements of two classes (the Hartman–Grobman theorem). If the topology changes between two phase flows with a continuous change in one parameter, one can say that a bifurcation occurred. Again this is a qualitative change, and not simply a matter of quantitative change, like scaling for instance. In the case of a two dimensional state space (defined by position and velocity coordinate), one theorem states that only fixed point (when stable corresponds to a stationary value in one coordinate in the physical space of the laboratory), limit cycle (a periodic evolution of one coordinate in physical space), and separatrix, are possible (the Bendixon–Poincaré theorem). A separatrix is a portion of the state space at which the trajectories diverge, because they are attracted in two opposite directions. Strong empirical evidence for the existence of this separatrix in the context of a reaction time task has being recently provided (Fink et al., 2009). Note that here topology encompasses a body of concepts and mathematics in the dynamical systems field, which may at times, differs significantly from the rather vague, but still intuitive and heuristic, use of the term by Bernstein and many of his followers. Armed with these basic topological considerations, one is able *a priori* to classify and then identify from empirical data certain elementary classes of movement, essentially the class of so-called discrete movement, and the class of so-called continuous movement. Moreover these classes of equivalence point to classes of models which could reproduce the observations. Recently these tools enabled the identification of primitives belonging respectively to the class of discrete and continuous movements in simple periodic movement (Huys et al., 2008), and in a reciprocal pointing task (Huys et al., 2010).

The distinct status of components and coordination implies the possibility that exchange between components do not affect, to some extent, the coordination. This property has been sometimes called “motor equivalence,” and was interpreted as a degree of abstractness of skills with respect to the specific implementation, for instance which limb is used. The famous example is that one can draw his/her name in the sand with the hand or with the foot. In the same vein, learning timing relations between two arms is transferred to two legs and vice versa (Kelso and Zanone, 2002). This “exchangeability” property, conservation of the macroscopic coordination ensuring the function despite components changes, is also termed sometimes termed degeneracy. Edelman and Gally (2001) proposed that degeneracy is ubiquitous at all levels of organization of biological systems: “unlike redundancy, which occurs when the same function is performed by identical elements, degeneracy, which involves structurally different elements, may yield the same or different functions depending on the context in which it is expressed.” Using group symmetry arguments, Golubitsky et al. (1999) (see Schöner et al., 1990) have shown that a whole class of specific neurophysiological systems can produce the same set of locomotion behaviors in various species, as long as they satisfy symmetric requirements. This means that the same behaviors can be achieved adaptively by a family of systems of interacting components. Recently, based on the same classification approach, Turvey et al. (2009) have shown that the way we measure distance by the use of our locomotor motion is determined by gait symmetry. Interestingly, Marey (1873) analyzed the coordination between two humans walking one closely following the other, and found that the single animal quadruped gaits are spontaneously adopted (see also Harrison and Richardson, 2009), hence the vast ensemble of group theoretic predictions for quadrupeds could be tested in a dyadic scheme in future experiments. Another validation of this abstract level of analysis came recently from the study of the movements of three people in a sport context, showing interacting dynamics well predicted by group theoretic arguments (Yokoyama and Yamamoto, 2011). More generally, those challenging current directions demonstrate again that climbing up the abstractness tree is not the ethereal grail of some remote scientist, but is practically useful, as it enables to model and predict natural processes despite an incomplete knowledge of various underlying components of the processes studied (Golubitsky and Stewart, 2006).

## INCURSIONS BETWEEN INDIVIDUALS

The relevance of movements involved in social behaviors is of course not restricted to humans. Fentress and colleagues analyzed quite completely the interaction between wolves in ritual’s fights (Moran et al., 1981). They found that sequences of clearly identified rotations and maintained distance between two animals formed a sort of syntax of relative motions, serving the recognition of roles played in the group. The way humans interact with others, the many purposes it may serve, is attracting a growing interest in behavioral sciences and neurosciences. The way we perceive other’s movement, how one can learn from observing another person, how we interpret others’ intentions, the role of seen gestures in communication, all of these issues relate to the interaction between individuals. However that in many cases, the question asked is about an observer of another person’s actions, and notice this is not interaction *per se*.

The way people interact jumped in the scope of coordination dynamics firstly to demonstrate how abstract the laws governing behaviors can be. Schmidt et al. (1990) ran a series of experiments asking two participants to swing periodically a pendulum while looking at each other. They actually found in this new problem all the hallmarks of bimanual coordination: bistable dynamics, bifurcation, critical fluctuations, and the like. This demonstrated that bimanual coordination laws are not the mere consequences of biomechanical or musculo-skeletal determiners, but can also be ruling when visual perception is the medium of the coupling. Therefore those laws are informational (Schöner and Kelso, 1988; Schöner, 1991; Kelso, 1994; Warren, 2006). Please note that in parallel several distinct frameworks have been developed and applied. One direction taken is to relate social coordination and communicative acts and related functions by addressing the so-called grounding problem. Imitation and early social interaction may offer an exit to the emergence of initial primitives required for the generative function of language. Another one is aimed at relating the framework presented here to the timely fashionable concept of embodiment of cognition (see, for example, Oullier and Basso, 2010). Furthermore one can cite the main concurrent approaches, in particular the theory of mind (TOM; Frith and Frith, 2003), but also the joint action theory (Knoblich and Jordan, 2003), or the extension of the internal model framework from individual’s movement to social interactive movements (Wolpert et al., 2003). In the two latter approaches prediction and anticipation of actions and decisions of others may be put forward as clearly complementary to a dynamical system account of social coordination. A dynamical system approach rests upon the instantaneous formalization provided by differential calculus, even seldom delayed differential equations have been used (see for a specific application to dyadic coordination Varlet et al., 2012). Arguably the challenge of anticipation in dynamical systems of human coordination, individual or social, which may minimally be defined as the state of the system under study at time (*t*) being determined (at least partly) by the state at time (*t* + tau), that is, the physicist abhorred causal influence of future onto present, remains to the best of our knowledge untouched. A throughout discussion about the theme of prediction is excluded here, however one may remember that dynamical systems are based on predictive assumption and a prediction objective, this corresponds to the program of formalizing determinism, originating with celestial mechanics. Put simply an evolution law acting upon a state space, be it for instance a large scale neural network underlying movement coordination, gives rise to a flow, a correspondence between a set of initial conditions and time evolving trajectories. Once the flow is given, a specific trajectory can be predicted given an initial condition; hence the future up to infinity is predicted. Please note that some authors may consider that the network, along with its dynamics (flow), *represents* the function for the organism, but this use of the word representation, Y stands for X, seems here abusive (see on this topic the recent work on neural population dynamics by Churchland et al., 2012). The trick is, based on an instantaneous step by step prediction, say differently the immediate future being a function of current state, the whole future is obtained. Now when noisy fluctuations are introduced, determinism is obtained at the level of densities probability functions, not of single realizations (Gardiner, 1990). This is the mathematical textbook saying, applications to human coordination of course require some wisdom to impose boundary conditions, and the formal infinity must be dropped, with good care like in any other applications, when one deals with actual individuals and experimental data. A third category of complementary frameworks correspond to the application of concepts and theorems from game theory, including Von Neuman’s minimax theorem and Nash’s equilibrium (Nash, 1950; see, for example, Braun et al., 2009, and references therein).

## TRACKING THE ONSET OF SYNCHRONIZATION BETWEEN PEOPLE

Next step was to turn the enquiry toward the core of social coordination dynamics. What could we learn from here? How much akin to synchronize we are, or state differently: how little is required to get our movement coordinated? Oullier et al. (2008) aimed at tracking in real time the onset of social coordination. To this end, the visual coupling between participants (i) was turned off, by keeping eyes closed, and then (ii) on, eyes open, while throughout the trial they were asked to oscillate their index finger “as if they had to do it the whole day.” Clearly, initially separated movements, in frequency and phase, rapidly converged after the visual exchange was on, to reach a synchronization state. Depending on initial condition at the onset of the visual exchange, in-phase or anti-phase pattern were adopted. In the third stage of the trial, the coupling was turned off by closing again the eyes, and the synchronized behavior dissolved, first the phases unlocked, then the frequencies slowly diverged, each participant continuing to move at a frequency close to the one adopted during the encounter. This simple experiment demonstrated that a collective behavior is very easily adopted, even without been instructed to voluntarily synchronize with the partner. The frequency of the intrinsic, spontaneously adopted, oscillatory movement during the eyes closed stage is sufficiently similar between two individuals to have a spontaneous mutual entrainment, giving rise to clear cut synchronization. Many features predicted by the theory of coupled oscillators were made quantifiable at the scale of the empirical observation, thanks to the possibility to manipulate the coupling medium, which is not so easily possible in interlimb coordination in one individual.

One question relevant to the newborn social neurosciences rapidly made its way once this paradigm was established. Given the frustrating difficulty to address a truly interactive situation between humans, it was still unresolved whether our brain activity was specific or not to those elementary epochs of synchrony between people. Building a dual electroencephalographic (EEG) recording set-up, and using a liquid crystal display to turn on and off the visual coupling from the movement of the partner, we were able to identify specific brain dynamics that correlated with effective synchrony (Tognoli et al., 2007). By comparing the EEG oscillatory content, at a very fine grain resolution, corresponding to epochs of spontaneous synchrony and epochs in which synchrony was not established while visual coupling was also present; we found systematic changes in the alpha band in right centro-parietal area. This study certainly gave the lead to the systematic investigation of interactive brains, which hopefully will further our definitions and understanding of various phenomena relevant to inter-individual behaviors, like resonance, mirroring, mimicry, symmetry breaking, agency and self-other discrimination, or attachment and rapport, to name but a few. One direction of research is to bridge the gap between the framework used in those studies and the mirror neurons and mirror network found in monkeys and humans. The now very famous discovery by Rizzolati and colleagues consisted in finding neurons in the premotor cortex responsive both when executing a specific action and when observing another individual executing the same action (Di Pellegrino et al., 1992). Neurons characterized by the same responsiveness were found in single cells recording in other areas, while later in humans brain imaging studies showed what was interpreted as a large scale mirror system, and more recently intra-cranial recordings in epilepsy patients revealed neurons with the same specific action dependency firing while executing or observing (see the research work by Mukamel et al., 2010). How this fits with the framework used here and the results obtained, or may be more efficiently one can ask what could be the role of the mirror neurons in mutual synchronization? The mirror neurons are implied in both perception, and in movement execution, thus naively one may assume they may support the coupling function between self-movement and the other’s proposed in the present approach. Some authors reject their mirroring function and replace it by a simple acquired perception–movement mapping, which could correspond to a coordinate frame change, like it is classically assumed in computational models of sensorimotor functions within an individual. Again the concept of a coupling can also account for this interpretation of the role of mirror neurons (please note that mapping and coupling here address two distinct levels of analysis). What remains is the action specific property of the mirror neurons. It is hard to conceive an action specific coupling, or the function of this coupling would be not to represent the observed movement but to select the proper intrinsic dynamics available in the repertoire of the observer matching the one observed. To address those questions, one may aim at extending the Tognoli et al.’s (2007) experiment to the coordination of two distinct movements. Minimally it seems readily feasible to address the coordination of one discrete and one continuous movement without losing the power and the current framework, but other type of differences between observed and executed movement patterns may have to be envisioned. To close this part, one cannot resist but evoking the studies showing how basic rhythmic behaviors, basically the actions implying a sensorimotor coupling with a periodic event in the environment, like the one introducing originally the present framework of coordination dynamics, are very likely to be originally acquired through social encounters (Kirschner and Tomasello, 2009; Wiltermuth and Heath, 2009).

## SYNCHRONY IS A PROCESS AND A SOLUTION

Synchronization is ubiquitous, rather well defined in terms of model and measurement, and its role in biology as long fascinated researchers (Wiener, 1948; Winfree, 1967; Kelso, 1995). But what could be its functional relevance, or ecological relevance? We can find very adaptive to step together when required, during metro rush hours of commuting, and this strong tendency can also become catastrophic (Moussaïd, 2011). In humans or animals, collective behaviors are suggested to increase sensory range (Couzin, 2009), and may serve visual attention. However collective behavior can prove difficult to relate to individual behavior (Gallup et al., 2012). Here is further food for thoughts coming from the study of brain networks dynamics. It is proposed that synchrony between localized emergent oscillations in the brain is a way to solve the binding problem of integrating segregated sensory features (Gray et al., 1989; von der Malsburg, 1994). Interareal coherence would serve information exchange between neurons and between neurons populations (Bressler et al., 1993). Clearly in this case synchrony reflects the exchange of information, because it necessarily depends upon coupling, given the asymmetry and noisy behaviors of components involved. But synchrony is likely to enable the proper timing of incoming flow of spiking activity relative to the most excitable times of local ongoing activities (Sejnowski and Paulsen, 2006; Senkowski et al., 2008). This way synchrony may strengthen the communication, making the receiving neurons more sensitive to incoming streams of spikes (Sejnowski and Paulsen, 2006).

There are other cases where the purpose of synchronization is intriguing. Consider a dancing couple. Sometimes one leads the other, but at the same time must keep up with the partner. Being an absolute beginner, I vividly remember how trying to teach me the basics of tango a female partner, by letting herself being guided, somewhat guided me to take over the lead. Here synchrony is a process, it has to be established, and a medium, in that it serves a purpose. By being selectively responsive to my leading movements, she reinforced my leading role. But who was leading?

I will draw now a provocative analogy. This type of leader–follower dynamics was seemingly operating in the dyad composed by a horse and his rider in simple seated trot dressage (Lagarde et al., 2005). We found evidence for an increase of synchrony between a highly trained rider and an equivalently highly trained horse when compared to the pair formed by an intermediate level rider and the same horse. What can we learn from this example? First take into account that this type of horse is extremely sensitive to any movement of the rider, through any surface contacts, mouth, legs, reins, stirrups, etc…. Obviously the aim of the rider is to control the horse, and we found evidence for a decrease of variability of the horse’s periods at maximal vertical extensions when ridden by the expert. That was precisely at those times that the intermediate rider synchronization to the horse vertical movement proved loose. In the same move the rider intend to get in sync with the horse and to controls the horse. Can we disentangle the two? What are the ingredients of this particular system? First the fact that the surface of contacts are bidirectional sensory interfaces, each one feels the other movements. Second that the rider cannot escape from adapting to the horse’s movements. Now if one aims at controlling, what matters are perturbing movements, that is, movements that introduce a change in the current state of affairs, a surprise, or in other words what can be considered as information. But those perturbing movements have to be intended, not the result of being somewhat passively shaken by the horse. For the rider to produce meaningful information a background of synchrony is welcome: by tightly following the horse the slightest deviation becomes relevant. The mutual coupling between the rider and the horse can be emblematic of the king of amplification process synchronization may offer, in a dyad of mutually perceiving and acting partners.

## FURTHER OUTSTANDING CHALLENGES

To close this short piece I will browse a short list of questions specific to social coordination waiting to be unveiled. The first issue relates to deciphering the specific information which is mediated by the coupling between individual’s movements. When one’s actions are determined by what he/she feels, hears or sees another person or a group of other person’s movements, is the information preferentially picked up specific to: single joint, pattern between joints, or end-effector (Varlet et al., 2011)? Second and related topic relates to the sensory modalities through which this coupling may be conveyed. It becomes increasingly clear that the ability to integrate or segregate the senses is key to adapted behavior, and that its disturbance may be conducive to a range of pathology. The senses within an individual presents asymmetries in their physiological properties and ecological uses that tailor their coordination dynamics, leading to favored or instead unstable behavior, in particular to define the boundaries between temporal and spatial fusion and segregation (Lagarde and Kelso, 2006; Lagarde et al., 2012; Zelic et al., 2012). What is the role of multisensory integration in the context of interpersonal coordination? Agency, motor resonance, out of body experience extended to another’s body (Farrer and Frith, 2002; Keysers and Gazzola, 2009; Blanke, 2012; Tajadura-Jiménez et al., 2012), all appear to rely on multisensory integration phenomena. Another promising challenge is to close the gap between the present framework and a game theoretic framework to account for similar elementary cooperative behaviors (Braun et al., 2009), and to provide a general taxonomy of such behaviors (see the propositions along those lines by Jarrassé et al., 2012), possibly by developing a framework as complete as the one employing topological equivalence presented above. Thirdly there is a need to understand whether or not the synchronization between two persons possesses a special status among social coordinative behaviors. We have briefly reviewed the current framework which describes in most details and most parsimonious way the formation of coordination phenomena. This framework in particular explain how a generic collapse of dimension arises each time a pattern is formed. Does this collapse of dimensions, to caricature, it could be sometimes said in dyadic coordination that one + one = one, have a particular meaning for humans? Is that the reason why such coordination episodes may affect the relation between two individuals, facilitating the communication for instance? This touches the question of the remnant of the coordination episode, its impact onto the individuals. This is key and at the same time may pose a difficult challenge. In **Figure 1** the general framework of synergetics and coordination dynamics is presented, and all the efforts to date have been put to propose the most general framework, yet able to generate empirically testable questions, explaining how coordination is formed. This entails in particular detailing how the system effective behavior changes from a high dimension to a lower dimension state. But consider now that the components interest us above all other things; this is plainly evident in a rehabilitation for social behavior context for instance. We really have in this case to analyze and model how the coordination level “feeds back” to the components. Oullier et al. (2008) found a sort of primitive social memory, the intrinsic preferred pace of the participants changed, at least transiently, after its spontaneous adjustment during the coordination stage with the partner thanks to mutual entrainment. This indicates a slow time scale dynamics operating at the level of the components. There are many straightforward ways in which this slow change could be introduced in the models, but one may wonder how deep the consequences would be for the whole framework, this feature of the components would prevent part of the elimination of dimension procedure which ensures the success.

Fourth and in relation to the third point, to contribute further to writing down the principles of interpersonal coordination, a clearer view about the role “symmetric” and “asymmetric” relations would be probably very informative (Boker and Rotondo, 2002). Here symmetric have to be understood as referring to the structure and intrinsic dynamics of the components, not the behavior. Symmetry between the intrinsic dynamics and the couplings of two individuals will give rise to synchronization, and possibly out of phase synchronization by half a period shift (Golubitsky and Stewart, 2006). However departure from symmetry can to some extent give also rise to behavioral synchronization. It would be very interesting to decipher the role played by asymmetric individual in a coordination task. Many measures ensuring the descriptions of those coordinations are relations, be it phase differences, or distances, thus are degenerate. As a consequence one cannot easily single out the contribution of the individual’s behavior within a pair. One way to overcome such limitation is to use asymmetric measures, the so-called causality measures, rarely used in behavioral sciences, for instance information transfer measures (De Guzman et al., 2005). These measures can potentially, in particular in the case of imperfect and dyadic coordination, indicate when one individual is more influenced by the other. To this aim, but in terms of experimental paradigm, another way to break the symmetry of measures and protocols is by use of the concept of the human clamp project, the virtual interacting partners (VPI; Kelso et al., 2009). By manipulating the parameters, intrinsic dynamics, and coupling function, of an artificial interacting agent, one may reveal hidden properties of the human actor. Kelso et al. (2009) judiciously reversed the coupling function within the artificial agent, to create a conflict between the human and the VPI and investigate novel phenomena. In the same vein, this insight about individual’s role within a pair can be examined by breaking the symmetry of movements, for instance by changing the moment of inertia of hand oscillated pendulum (Varlet et al., 2012), but more radical differences can be introduced. A change in one such parameter stands either for a quantitative change, and this has been investigated in within individual bimanual coordination and between individuals (Varlet et al., 2012), but also to a more important qualitative change, as presented in the course of this paper.

To conclude, and coming back to the introduction, it may be time that we depart from symmetric coordination, to understand further how we evolve in and out of perfect dyadic synchronization. As stated in the introductory example, observing eyes are also perturbing. Coupling is explicitly interpreted and dealt with by mathematicians as a perturbation of intrinsic (isolated) dynamics of the components, hence the presence of the observer offers a source of potential information creation (Boker and Rotondo, 2002). The mirror will not tell you much about yourself, but another person, by definition different, may do so.

## Conflict of Interest Statement

The author declares that the research was conducted in the absence of any commercial or financial relationships that could be construed as a potential conflict of interest.

## Footnotes

1 It is fair to remark that *being* is identified here with *public statements*, in a sense denoting a very behavioral posture which could rise a whole set of criticisms, and that the separation self~others is specifically generated by the institutional individual responsibility belonging to the act of formulating public statements.
